# Analysis of Parenting Attitude Types and Influencing Factors of Korean Parents by Using Latent Transition Analysis

**DOI:** 10.3390/ijerph18147394

**Published:** 2021-07-10

**Authors:** Hanna Lee, Jeong-Won Han

**Affiliations:** 1 Department of Nursing, Gangneung-Wonju National University, Wonju-si 26403, Korea; hannalee@gwnu.ac.kr; 2 College of Nursing Science, Kyung Hee University, Seoul 02447, Korea

**Keywords:** child, parents, parenting, longitudinal studies

## Abstract

This study aimed to classify the latent class of parenting attitude for parents with preschool children and school-age children, identify the pattern of transition in the type of parenting attitude over time, and determine the influencing factors associated with the transition. A total of 1462 households were the subjects of this longitudinal study that used latent profile analysis, latent transition analysis, and logistic regression analysis. The parenting attitude in the preschool year was classified into a model of three latent classes of ‘parent uninvolved’, ‘maternal authoritative and paternal authoritarian’, and ‘maternal authoritarian and paternal authoritative’, and the parenting attitude in the school year was classified into a model of four latent classes of ‘parent weak uninvolved’, ‘parent strong uninvolved’, parent authoritative’, and ‘maternal authoritarian and paternal authoritative.’ All latent class subjects with preschool children showed an attitude transition to maternal authoritarian and paternal authoritative when their children were in school years. It was confirmed that a mother’s depression and father’s parenting stress were the most influential factors in the parenting attitude transition. This study lay in identifying the patterns of parenting attitude and the transition in attitude according to the developmental stage of children.

## 1. Introduction

Home is the first environment an individual encounters after birth, and the home environment has a lasting impact on the individual throughout their lifetime. At home, parents provide parental care for children, and parenting attitude has been emphasized as a key factor influencing children’s social and emotional development [1,2]. Parenting attitude is a disposition or a response style in child-rearing to promote the growth and development of children. It refers to a general attitude exhibited when parents raise their children [3]. At the time of transition from preschool to school age, children learn and form and maintain peer relationships in their school based on their interactions with parents; thus, parenting attitude has a direct impact on children’s development [4]. Consequently, the type of relationship the child forms with his/her parents and the parenting attitude of the parents toward their child may have a significant impact on the behaviors of school-age children, such as relationships with teachers and schoolmates [5].

Different types of parenting attitudes have been proposed in previous studies. Baumrind [6] classified parenting styles into authoritative, permissive, authoritarian, and uninvolved, based on the two dimensions of parental support and parental control. Authoritative parents teach the importance of social values while respecting the individuality of their children. They exercise strict control when necessary. This type of parent values verbal communication with their children. In contrast, authoritarian parents value control or unconditional obedience, punish their children when principles are violated, and show a cold attitude towards their children. Permissive parents value self-expression and self-regulation, consider themselves as supporters, do not punish their children, and are relatively warm in their attitude. Uninvolved parents exercise very limited acceptance and control of their children, providing only minimal attention. Moccoby and Martin classified parenting styles into four types based on two dimensions: parental demandingness (control, supervision, maturity) and parental responsiveness (affection, permission, involvement) [7]. The authoritative attitude is child-centered and acknowledges and accepts the child’s needs and wishes, and the authoritarian attitude is a dictatorial attitude that restricts the expression of desires and oppresses children who challenge parental authority. Permissive attitudes are affectionate, responsive, and have little control over the child’s behavior, while neglectful attitudes are attitudes that reject or ignore the child’s needs and provide no guidance or discipline. Williams and Wahler [8] classified parenting styles into three types: authoritative, authoritarian, and permissive.

Parenting styles have an impact on the overall aspects of a child’s life. A loving attitude and parental guidance contribute to the development of empathy and prosocial behavior of the child, whereas coercive, punishment-oriented discipline and an uninvolved attitude lower the emotional happiness of the child. This leads to the child showing signs of aggression. In addition, such attitudes also impede the development of social skills, resulting in rebellious and problematic behaviors in a child [9]. Galambos et al. [10] investigated the trajectories of change in externalizing and internalizing problems in children for three years. They found that when parents had parenting styles of firm control and supervision of the behavior and life of their children, the children showed fewer problem behaviors. Contrarily, when parents were overly interfering or had a parenting style with no affection, this had a negative impact on the problem behaviors of the child. Positive parenting attitudes influence the self-directed learning ability of children, enhance their social skills, and adaptability to school life [5,11,12]. In recent years, as family interactions with parents have increased due to limitations in children’s external and social activities due to the COVID-19 pandemic, there has been increasing emphasis on the importance of parenting attitude. In addition, considering that children’s social and cyber delinquency and aggression are also increasing compared to the past, an in-depth investigation of parenting attitude is needed, and when necessary, appropriate intervention from experts is required.

Parenting attitude is not a simple concept but is formed by a variety of determinants, such as parenting stress perceived by parents [11,13], marital conflict, marital satisfaction [14], and depression [15,16,17]. In particular, parenting stress perceived by parents is a decisive factor in determining parenting attitude [18], and as the level of parenting stress increases, the positive parenting attitude toward the child decreases, and the negative parenting attitude tends to increase [13]. In addition, previous studies have confirmed that parenting attitude is negatively affected when the mother and father have marital conflict or low marital satisfaction [14,19]. Depression is one of the most commonly reported psychological disorders among mothers raising preschool children, and maternal depression has been reported to show parental withdrawal toward the child and negatively influence parenting attitude [20].

In particular, in modern society, with more women involved in breadwinning and other social activities, the proportion of fathers’ involvement in child rearing has increased; therefore, inter-parental interactions affect parenting attitude. Furthermore, since attitude is not static but variable by the environment of each person, longitudinal studies are required to investigate parenting attitudes rather than individual or cross-sectional approaches [11]. However, prior studies on parenting attitude have primarily investigated the maternal side or adopted a variable-centered approach. The variable-centered approach is useful when determining which predictive variable explains more of the variation in the reference variable [21]. However, with the variable-oriented approach, which mainly looks into the average relationship in the population, it is difficult to examine how the various factors that comprise the parenting attitude are combined within an individual and affect their children. In this regard, it is necessary to examine parenting attitude by applying a latent profile analysis (LPA) with a person-centered approach. LPA can reasonably determine the number of latent classes using a model-based method and identify classes with common attributes or with similar relationships between attributes [21]. In addition, for the investigation of parenting attitude, the change over time and the approach in consideration of the change is important rather than the parenting attitude at a specific time. In the case of prior studies with cross-sectional data, it is difficult to identify how parenting attitude changes with the age of the children, that is, how it changes over time [18,22].

In the West, children naturally experience an environment and culture that respects individual privacy and different opinions on their growth, but in the East, filial duty and respect for seniority are valued, and parental care plays a central role until the child becomes an adult and leads his/her independent life. In the Western view, parents value individual interests and abilities of their children, while Korean parents value the understanding and interests of the group to which their children belong. After all, parents in western culture focus on raising their children as autonomous human beings where the expression of their emotions is important. Parents in eastern culture focus on raising their children with the ability to control their desires and emotions. Compared to the past 10 years, in terms of the parenting culture perceived by parents in South Korea, educational fervor is still, or rather increasingly, a powerful keyword, even to the point of becoming excessive, and the parenting attitude is changing as a consequence. Therefore, in order to promote a more extensive understanding of parenting attitude in South Korea, this study aimed to explore the pattern in the latent class of parenting attitude when the child is in preschool years and at the age of 7, the start of the school year. In addition, by examining the transition pattern in the latent class of parenting attitude at respective points of time using latent transition analysis (LTA), the possibility of change in parenting attitude over time and the stability of the change were investigated, and the relevant implications have been presented.

## 2. Materials and Methods

### 2.1. Participants

The subjects of this study were parents who participated in the 5th (2012)–8th (2015) survey of the Panel Study on Korean Children (PSKC). This was a longitudinal study using latent transition analysis (LTA). It included 1462 households where both the parents participated in the PSKC survey of the applicable period and were the final subjects of this study. Currently, in the PSKC, parenting attitude is measured only up to the 8th round year, and the 8th year is the first year in which a child goes to school in South Korea. Since this is the time when children undergo a transition from preschool to school years, they learn, form, and maintain peer relationships in a school society based on their interactions with parents, and thus, parenting attitude plays a critical role at this time [4]. The data of the 5th–8th years were finally selected from the PSKC data. In a simulation study on determining the optimal number of latent classes by applying latent class analysis (LCA) [23], the type I error, power, and fit of the model were analyzed according to three different sample sizes of 200, 500, and 1000. The results showed that sample sizes of 500 and 1000 were a better fit compared to the sample size of 200. In addition, although there is a difference in the required size of samples depending on the structure of the latent profile model, such as the number of sub-variables used in LCA and the number and size of latent classes, the fit of the model improved when sample size was increased. Therefore, in this study, the number of samples, which was 1462 households (1462 mothers, 1462 fathers), could be considered to be the number of samples appropriate for LTA. Participants in this study characteristic were as follows: The mean age of the fathers was 40.38 years, and that of the mothers was 38.02 years. Examining the educational level, the education level of fathers consisted of 386 (26.4%) high school graduates, 262 junior college graduates (17.9%), and 814 (55.7%) university graduates or higher. The education level of mothers consisted of 433 high school graduates (29.6%), 371 (25.4%) junior college graduates, and 658 (45.0%) university graduates or higher. The residential areas of the subjects consisted of 602 (41.2%) in large cities, 212 (14.5%) in eup/myeon (towns/villages), and 648 (44.3%) in small and medium-sized cities. The average household income was $4193.14, and 452 (30.9%) were working mothers. The occupation types of fathers included 716 (49.0%) professionals and office workers, 189 (12.9%) machine operation and assembly workers, 174 (11.9%) craft and related trade workers, and 164 (11.2%) sales workers (Table 1).

### 2.2. Measurement

#### 2.2.1. Parenting Attitude

For assessment of parenting attitude, the tool developed by the PSKC research team, which is based on the findings of Cho et al. [24], was used, which consists of 12 questions in total. It is composed of two sub-areas, warm parenting attitude and controlling parenting attitude, and each question is rated on a 5-point Likert scale, and the higher the score, the higher the degree of the sub-area. The PSKC research team did not present the reliability of the parenting attitude questions, but in this study, the reliability (Cronbach’s alpha) of the tool in the 5th and 8th years of the survey were 0.78–0.81 and 0.82–0.83, respectively.

#### 2.2.2. Parenting Stress

For assessment of parenting stress, from the sub-factors of the scale developed by Kim and Kang [25], the parts on the burden and distress from performing the parental role were extracted, and a total of 11 questions were confirmed through a preliminary study in 2007 by PSKC researchers. These 11 questions were used as the instrument in this study. Each question was rated on a 5-point Likert scale, and the higher the score, the higher the parenting stress. In the study by Kim and Kang [25], the reliability (Cronbach’s alpha) of the tool was 0.88. However, in this study, Cronbach’s alpha was 0.86–0.88 in the 5th year survey.

#### 2.2.3. Marital Conflict

To assess marital conflict, the Marital Conflict Scale developed by Markman et al. [26] was used, and to ensure consistency in the questionnaire, the PSKC researchers composed the questions using a 5-point Likert scale and eight questions in total; the higher the score, the higher the level of conflict perceived by parents. In the study by Markman et al. [26], the Cronbach’s alpha was 0.92; in this study, Cronbach’s alpha was 0.91–0.92 in the 5th year survey.

#### 2.2.4. Marital Satisfaction

To assess marital satisfaction, an instrument developed by Chung [27] was used, which consists of 4 questions in total, and the PSKC researchers composed the questions with a 5-point Likert scale in the 5th survey and a 4-point Likert scale in the 8th survey. The higher the score, the higher the marital satisfaction perceived by the parents. In the study by Chung [27], Cronbach’s alpha was 0.94. In this study, Cronbach’s alpha was 0.92 in the 5th year survey.

#### 2.2.5. Self-Esteem

To assess self-esteem, a scale developed by Rosenberg [28] was used, which consists of a total of 10 questions rated on a 5-point Likert scale, the higher scores indicating higher self-esteem. In Rosenberg’s study, the reliability of the tool was represented with a Guttman scale coefficient of a reproducibility of 0.92; in this study, the reliability of the tool was represented with Cronbach’s alpha at 0.85–0.86 in the 5th year survey.

#### 2.2.6. Depression

To assess depression, a scale developed by Kessler et al. [29] was used. It consists of a 5-point Likert scale with a total of 6 questions, and the higher the score, the higher the degree of depression. In the study by Kessler et al. [29], Cronbach’s alpha was 0.89, whereas in this study, Cronbach’s alpha was 0.91 in the 5th year survey.

### 2.3. Data Collection

The data provided through the PSKC website (http://panel.kicce.re.kr/kor/publication/02.jsp) (accessed on 1 May 2021) were used for this study. In the data used, the codes (name, resident registration number, etc.) for personal identification were redacted. The Korea Institute of Child Care and Education recruited 2562 households for the preliminary sample of the PSKC survey, of which 2150 households with newborns were extracted as the final sample. For the PSKC samples, a stratified multistage sampling method was used. In the first stage, medical institutions that provide the service of delivering newborns were selected; in the second stage, households with newborns with childbirth in the selected medical institution were extracted as preliminary samples; in the third stage, from the preliminary sample households, the households that were willing to participate in the panel study were finally selected as the samples.

### 2.4. Ethical Considerations

The study was conducted upon exemption from review by the Institutional Review Board (GWNUIRB-R2021-42).

### 2.5. Data Analysis

To analyze the general characteristics of subjects, descriptive statistics, and the differences in parenting attitude between the classified latent types, SPSS 24.0 (Data Solution Inc., Seoul, Korea) was used to obtain frequency, percentage, mean, and standard deviation, and an ANOVA was performed. In this study, the latent class of parenting attitude was explored using latent profile analysis (LPA), and the transition pattern of latent class and factors affecting the transition were investigated by applying latent transition analysis (LTA). LTA is a type of latent Markov model, and it is a variation of latent class analysis that is suitable for examining patterns of changes in a latent class using longitudinal data or identifying factors affecting the change [30]. In addition to the ratio of each latent class in the latent class analysis and the item response probability, which is the probability of each latent class’s response to a specific item in a specific category, LTA is a method designed to enable the estimation of transition probability, the probability of each latent class of previous timepoint transitioning to each latent class of the next timepoint. Transition probability refers to the probability of transition of each latent class estimated at time t − 1 into a specific latent class at time t. If the data are measured at time t, the estimation of the transition probability from time t − 1 is possible. To determine the number of latent classes, information index and a model comparison test were used. Representative information indexes included Akaike Information Criterion (AIC) [31], BIC (Bayesian Information Criterion) [32], SABIC (Sample-Size Adjusted BIC) [33], LMR LRT (Lo-Mendell–Rubin Adjusted Likelihood Ratio Test) [34], and BLRT (parametric bootstrap likelihood ratio test) [35]. Through the test results of (k − 1) latent class models and k latent class models, the final model was selected. If the p-value was not within the set significance level, (k − 1) latent class models were selected, and if the value was within the significance level, k latent class models were selected. Determining the optimal number of classes in the mixed model is one of the key issues, because the interpretation and inference of the results of the study are conducted according to the number of classes. There is no consensus criterion for determining the optimal number of classes, and discussion and research on the appropriate goodness-of-fit index of information are underway. In this study, the goodness-of-fit index of information was comprehensively reviewed considering the above points, and the final model was selected by considering the possibility of interpretation and other factors. In this study, Mplus 7.4 [36] was used for LTA. LTA estimates the probability that the type of parenting attitude at the previous time will change to another type at the next time, since the parenting attitude type can be different for each time point [30]. Logistic regression analysis was performed to analyze the factors influencing the transition of parenting attitude type.

## 3. Results

### 3.1. Latent Classes by Timepoint

**Figure 1:**
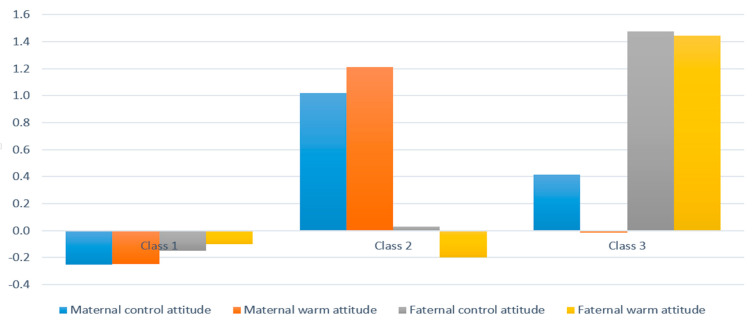
Latent class of child-rearing attitude (5th wave).

In the case of parenting attitude for preschool children (children’s age: 4 years old), the mother’s controlling parenting attitude scored 20.22 ± 3.11 points on average, the warm parenting attitude scored 21.97 ± 3.24 points, the father’s controlling parenting attitude averaged 19.48 ± 3.61 points, and the warm parenting attitude averaged 21.24 ± 3.55 points. As a result of latent profile analysis (LPA) on parenting attitude when a child is in the preschool year, in the analysis of goodness-of-fit index of information, the values of AIC, BIC, and BIC (SABIC) were lower when the number of latent classes was three, compared to the number of latent classes at one or two. When the number of latent classes was four in the model, the values of AIC and BIC were lower, but the value of LMR LRT was 0.051, thus indicating that the result was not significant at the significance level of 0.05. Based on this result, a model with three latent classes was confirmed to be the optimal model for the analysis of parenting attitude in the preschool year (Table 2) (Figure 1). When examining the characteristics of the final classified classes, Class 1 had an uninvolved parenting attitude, in which both the controlling attitude and warm attitude scored low in both parents, and it was named ‘Parents (P)_uninvolved. A total of 1122 households were included in this class out of the total subjects. In Class 2, mothers showed an authoritative parenting attitude, in which both the controlling attitude and warm attitude scored high, whereas, fathers showed an authoritarian parenting attitude, in which the controlling attitude scored high and the warm attitude scored low, and this class was named ‘Mother (M)_authoritative and Father (F)_authoritarian’. A total of 232 households were included in Class 2. In Class 3, mothers showed an authoritarian parenting attitude, in which a controlling attitude scored high and a warm attitude scored low, whereas fathers showed an authoritative parenting attitude, in which both the controlling attitude and warm attitude scored high. This class was named ‘M_authoritarian and F_authoritative’, and 108 households were included in this class out of the total subjects.

**Figure 2:**
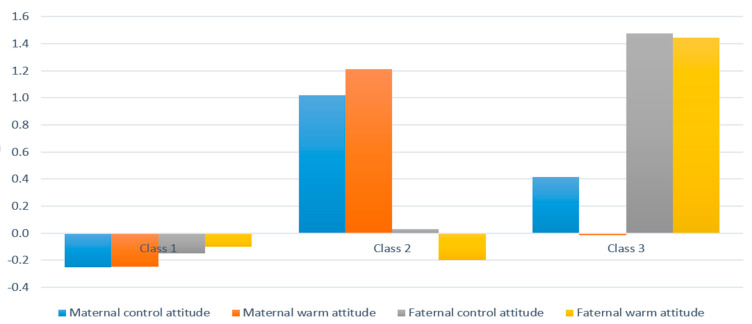
Latent class of child-rearing attitude (8th wave).

In the case of parenting attitude for school-age children (children’s age: 7 years old), the mother’s controlling parenting attitude scored 21.03 ± 2.78 points on average, the warm parenting attitude scored 22.06 ± 3.32 points, the father’s controlling parenting attitude averaged 20.52 ± 3.47 points, and the warm parenting attitude averaged 21.58 ± 3.73. As a result of LPA on parenting attitude when a child was in the school year, in the analysis of the goodness-of-fit index of information, the values of AIC, BIC, and BIC (SABIC) were lower when the number of latent classes was four, compared to the number of latent classes at one, two, or three. When the number of latent classes was five in the model, the values of AIC, BIC, and BIC (SABIC) were lower, and the values of LMR LRT and BLRT were significant at the significance level of 0.05, compared to the case where the number of latent classes was four in the model, but the explanation of the class type was not clear. Based on this result, a model with four latent classes was confirmed to be the optimal model for the analysis of parenting attitude in the school year (Table 3) (Figure 2). When examining the characteristics of the final classified classes, Class 1 had an uninvolved parenting attitude, in which both the controlling attitude and warm attitude scored lower compared to other classes in both parents. This class was named ‘P_weak uninvolved’, and 468 households were included in this class out of the total subjects. In Class 2, both the controlling attitude and warm attitude scored higher compared to other classes in both parents. This class was named ‘P_strong uninvolved’, and 90 households were included in this class. In Class 3, both the controlling attitude and warm attitude scored high in both parents, and this class was named ‘P_authoritative’. A total of 177 households were included in Class 3. In Class 4, mothers showed a weak authoritarian parenting attitude, in which controlling attitude scored high and warm attitude scored low, whereas, fathers showed an authoritative parenting attitude, in which both the controlling attitude and warm attitude scored high. This class was named ‘‘M_authoritarian and F_authoritative’, and a total of 727 households were included in this class out of the total subjects.

### 3.2. Transition Phenomenon of Parenting Attitude

**Figure 3:**
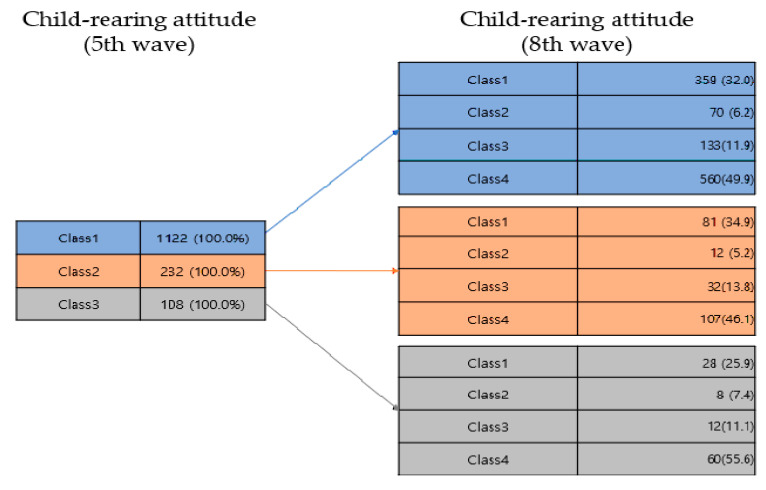
Transition analysis of child-rearing attitude.

Table 4 and Figure 3 show the results for the patterns of parenting attitude transitions. In this study, all parents with latent classes of Class 1, Class 2, or Class 3 when their children were in the preschool year transitioned to the ‘M_authoritarian and F authoritative’ (Class 4) type the most when their children were in the school year.

### 3.3. Factors Influencing the Transition of Parenting Attitude Type

In this study, logistic regression was performed to examine the factors influencing the transition of parenting attitude type. The results of analyzing the factors influencing the transition to ‘M_authoritarian and F_authoritative’ (Class 4), the type which showed the most transition into in the school year from the parenting attitude types in the preschool year can be summarized as follows. The higher the depression of mothers of preschool children, the higher the transition probability of ‘P_uninvolved’ type to ‘M_authoritarian and F_authoritative’ in the school year (OR = 1.23, 95% CI = 1.01–1.45). The higher the parenting stress of fathers of preschool children, the higher the transition probability of ‘M_authoritative and F_authoritarian’ to ‘M_authoritarian and F_authoritative’ in the school year (OR = 1.77, 95% CI = 1.36–12.16). The higher the depression of mothers of preschool children, the higher the probability of maintaining ‘M_authoritarian and F_authoritative’ as it was in the school year (OR = 2.28, 95% CI = 1.24–3.42).

## 4. Discussion

In this study, the latent class of parenting attitude was classified through latent transition analysis (LTA) to identify the characteristics of each class, and the transition patterns of parenting attitude were investigated. Based on the findings of this study, the following implications are outlined:

First, the parenting attitude in the preschool year was classified into a model of three latent classes of ‘P_uninvolved’, ‘M_authoritative and F_authoritarian’, and ‘M_authoritarian and F_authoritative’, and the parenting attitude in the school year was classified into a model of four latent classes of ‘P_weak uninvolved’, ‘P_strong uninvolved’, P_authoritative’, and ‘M_authoritarian and F_authoritative.’ This result is similar to the result reported by a previous study conducted on Belgian parents who were 8–10 years old [37], in which parenting style was classified into four types: authoritative, positive authoritative, authoritarian, and uninvolved. The result is also similar in part to the result reported in the US by a cluster analysis of mothers’ parenting attitudes, which showed the classification into six classes [38]. However, since most of the previous studies have assessed parenting attitude at a specific time point, it is difficult to determine and compare the parenting attitude type with the results of this study. However, the results of this study show that the types of parenting attitudes vary with the age of the child, and the types can change over time. Considering that parenting attitude can change according to various psychosocial factors, and that in the period of transition from preschool years to school years, the children undergo rapid changes in physical, psychological, and social aspects among the developmental stages of children [39], it is important that the experts pay attention to parenting styles from the preschool years. Thus, when any problem arises at each time point, they can select appropriate intervention strategies.

Second, in this study, ‘P_uninvolved’ was the parenting type that accounted for the highest proportion of parents of preschool children, and this was a class that scored low in both the controlling attitude and warm attitude in both parents. Controlling parenting behavior refers to firm teaching of manners and rules to the child, imposing restrictions on the child’s behavior and punishing or imposing restrictions in case of a child’s wrongdoing [6]. It was inferred that the reason for low scores in the controlling attitude in parents of preschool children was that since the children were young, they had difficulties in controlling emotions. However, with little development in language skills, they would experience difficulties in communication, thereby causing difficulties for parents to teach the child strict rules and imposing restrictions on their behavior. A warm parenting attitude refers to a parenting behavior in which parents express affection and interest toward the child, recognize the child’s independence, and have a high level of communication with the child [6]. It was inferred that the reason for low scores in the warm attitude of parents of preschool children was that it was difficult for the parents to recognize or grant the independence of the child in this stage.

Third, in this study, the type of parenting attitude to which the most transition occurred was ‘M_authoritarian and F_authoritative’. In particular, in the case of mothers of school-age children, the characteristics of controlling parenting behavior scored high in comparison to other types of attitudes. This result is similar to the result of a parenting attitude study of Korean mothers [40], in which the firm and strict parenting type accounted for the highest proportion at 39.4%. It is also similar to the result of a parenting style study of Chinese mothers [41], in which Chinese mothers showed a high level of controlling behavior. This indicates that in Eastern culture, mothers show a high level of interest in and protection for school-age children. A controlling parenting attitude refers to a behavior in which parents pay attention and control their children’s behavior, including continuous tracking and monitoring over where the child is, what he or she is doing, and whether he/she is adapting smoothly [42]. In the Western culture that places importance on individual independence, parental control is perceived as negative, but in the Asian culture, parental control is regarded as a necessary interference, that is, “not being indifferent”, and therefore, controlling parenting styles have been reported to have positive effects in some cases, depending on the cultures [43]. Therefore, as can be seen from the results of this study, it is thought that Korean mothers discipline their children in the period of transition to the school year so that the child can comply with the expectations and customs of society. Hence, the mothers scored high in controlling attitude. However, studies on adolescents have shown that an excessive controlling attitude causes negative emotions in children and increases depression, compulsion, and aggression [43]. There is a need to place emphasis on parenting education so that parents can show an authoritative parenting attitude in which the parents explain to the child the reason for the discipline, while showing affection toward the child. In addition, in the ‘P_authoritative’ class, which could be regarded as the most positive class of parenting attitude, only the parents of 177 households were included in the school-age year. Authoritative parenting behavior may have a significant impact on self-esteem, psychology, sociality, and academic performance. Moreover, it has been reported that the parenting style that grants and supports the autonomy of children enhances the self-esteem of the child, lowers depression, and increases satisfaction in life, thus having a positive impact on the sense of psychological stability [44]. In particular, the time of transition from preschool to school is the period that plays a critical role in the physical and psychological adaptation and development of the child, and in this stage, an authoritative parenting attitude has a significant impact on the child. Therefore, considering the positive direction of development for the child, parenting education needs to be designed and provided for parents to develop an authoritative parenting attitude.

Fourth, examining the factors that influence the transition to ‘M_authoritarian and F_authoritative’, the class to which transition occurred the most in this study, the depression of mothers of preschool children (P_uninvolved, M_authoritarian and F_authoritative) was identified as an important factor. This is consistent with the results of a previous study [24] that depressed mothers have a high level of fear and anxiety about mistakes, so they may show a disposition to suppress their children without granting autonomy while raising their children. As can be seen from the results of another study that reported that parental depression has a negative impact on parents’ psychological and emotional aspects and that unstable psychological and emotional states of the parents cause inappropriate parent–child interactions [17], the psychological state of parents serves as a critical factor in determining parenting behavior. Therefore, it is necessary to promote the establishment and implementation of parental education programs, which are easy to access, to address the psychological problems of parents. To this end, health care providers, such as public health centers and community health centers, should proactively screen parents who have a general level of depression and not just those with clinical depression, promote more programs including these parents, and endeavor to provide improvements in support of these programs. In addition, the higher the parenting stress of fathers of preschool children, the higher the transition probability from the ‘M_authoritative and F_authoritarian’ class to the ‘M_authoritarian, F_authoritative’ class in the school year. This result is consistent with a previous study that showed that the mother’s controlling parenting attitude is affected by the rate of change in the father’s parenting stress [11]. This result is similar to another study, which reported that father’s involvement in parenting had a negative correlation with mother’s parenting stress [45]. In particular, the excessive parenting stress of the father causes the mother to feel skeptical about the parental role and show withdrawn or excessive parental behavior, leading to a negative impact on the psychological and emotional aspects of the child [13]. Therefore, it will be necessary to develop programs, such as the establishment of a self-help class, that can resolve the parenting stresses of both parents.

## 5. Conclusions

The significance of this study lay in its identification of the latent class of parenting attitude through latent transition analysis (LTA), investigating the pattern of the profile and transition pattern of parenting attitude changes for each time point using longitudinal data and demonstrating that the parenting attitude change differs from person to person due to various influencing factors. The results of this study showed that the latent class with the most transition among parenting attitude types was ‘M_authoritarian and F_authoritative’, and it was confirmed that a mother’s depression and father’s parenting stress were the most influential factors in the parenting attitude transition. Therefore, accurate assessment of the changing patterns of parenting attitude is instrumental for the growth and development of children. Moreover, it is necessary to establish community facilities and deploy experts who can regularly check and examine the psychological state of parents. In addition, if an intervention program can be developed and implemented to reduce parenting stress, it is expected to be useful and effective in improving parenting attitude as well as reducing parenting stress. This study has been significant in investigating the change in parenting attitude with longitudinal data; however, only the change patterns at the two time points of the ages of 4 and 7 of the children were examined. Thus, further studies are required to investigate the transition pattern of parenting attitude at various time points.

## Figures and Tables

**Table 1:** Participants’ characteristics.

Variables Category		*n* (%)	
Father’s age (yr)	<36	148	10.1
(M ± SD = 40.38 ± 3.81)	36–40	612	41.8
	>40	702	48.1
Mother’s age (yr)	<36	526	36.0
(M ± SD = 38.02 ± 3.76)	36–40	531	36.3
	>40	405	27.7
Father’s education	Under high school	386	26.4
	College	262	17.9
	Over bachelor’s degree	814	55.7
Mother’s education	Under high school	433	29.6
	College	371	25.4
	Over bachelor’s degree	658	45.0
Residential areas	Large cities	602	41.2
	Eup/myeon (towns/villages)	212	14.5
	Small and medium-sized cities	648	44.3
Father’s occupation	Professionals and office workers	716	49.0
	Machine operation and assembly workers	189	12.9
	Craft and related trade workers	174	11.9
	Sales workers	164	11.2
	Others	219	15.0
Mother’s occupation	Manager or white collar job	452	30.9
	House wife and other	1010	69.1
Family income ($)	<4000	734	50.2
(M ± SD = 4193.147)	≥4000	728	48.8

M = mean, SD = Standard deviation.

**Table 2:** Model fit of latent profile analysis of child-rearing attitude (5th wave).

Model	AIC	BIC	saBIC	LMR-LRT	BLRT	Latent Class Distribution Rate (%)			
						1	2	3	4
1 profile	30,835.02	30,877.33	30,851.91	-	-	100.0	-	-	-
2 profile	30,807.09	30,875.83	30,834.53	0.079	<0.001	92.4	7.6	-	-
3 profile	30,791.62	30,786.80	30,729.62	0.039	<0.001	76.7	15.9	7.4	-
4 profile	30,766.40	30,788.01	30,714.95	0.051	<0.001	0.8	5.9	78.2	15.1

AIC = Akaike Information Criterion, BIC = Bayesian information criteria, saBIC = Sample-size adjusted Bayesian information criteria, LMR LRT = Mendell–Rubin likelihood ratio test, BLRT = Bootstrapped likelihood ratio test.

**Table 3:** Model fit of latent profile analysis of child-rearing attitude (8th wave).

Model	AIC	BIC	saBIC	LMR-LRT	BLRT	Latent Class Distribution Rate (%)				
						1	2	3	4	5
1 profile	30,608.04	30,650.346	30,624.933	-	-	100.0	-	-	-	-
2 profile	29,367.24	29,435.986	29,394.689	<0.001	<0.001	36.9	63.1	-	-	-
3 profile	28,664.67	28,759.847	28,702.667	0.034	<0.001	7.5	49.5	43.0	-	-
4 profile	27,945.90	28,067.517	27,994.453	0.045	<0.001	32.0	6.2	12.1	49.7	-
5 profile	27,364.67	27,512.723	27,423.776	0.032	<0.001	3.6	23.5	30.0	35.4	7.5

AIC = Akaike Information Criterion, BIC = Bayesian information criteria, saBIC = Sample-size adjusted Bayesian information criteria, LMR LRT = Mendell–Rubin likelihood ratio test, BLRT = Bootstrapped likelihood ratio test.

**Table 4:** Transition analysis of child-rearing attitude.

5th Wave			8th Wave			
*n* (%)			Class 1 *n* (%)	Class 2 *n* (%)	Class 3 *n* (%)	Class 4 *n* (%)
Class 1	1122 (100.0)	→	359 (32.0)	70 (6.2)	133 (11.9)	560 (49.9)
Class 2	232 (100.0)	→	81 (34.9)	12 (5.2)	32 (13.8)	107 (46.1)
Class 3	108 (100.0)	→	28 (25.9)	8 (7.4)	12 (11.1)	60 (55.6)

5th wave: Class 1 = Parents_uninvolved, Class 2 = Mother_authoritative and Father_authoritarian, Class 3 = Mother_authoritarian and Father_authoritative, 8th wave: Class 1 = Parents_weak uninvolved, Class 2 = Parents_strong uninvolved, Class 3 = Parents_authoritative, Class 4 = Mother_authoritarian and Father_authoritative.

## Data Availability

No new data were created or analyzed in this study. Data sharing is not applicable to this article.
